# Supplementary material for: Cloud BioLinux: pre-configured and on-demand bioinformatics computing for the genomics community
Source: BMC Bioinformatics. 2012 Mar 19;13:42. doi: 10.1186/1471-2105-13-42 (PMC3372431; doi:10.1186/1471-2105-13-42)
Supplement: Additional file 1 — Supplementary 1 Cloud BioLinux software documentation in the form of a mini, self-contained website. Users need to download and uncompress the .zip file, and open through a web browser the "index.html" file available on the main directory. (ZIP 1823 kb). [file 1471-2105-13-42-S1.ZIP › Cloud-BioLinux-Package-Documentation/docs/rpsblast.html]

Bio-Linux Software Documentation Pages

Back to search form

## rpsblast

|  |  |
| --- | --- |
| Name | rpsblast |
| Description | rpsblast (Reverse PSI-BLAST) searches a query sequence against a database of profiles, or score matrices, prepared by copymat, producing BLAST-like output.  **rpsblast** on Bio-Linux is one of the programs distributed along with the old blastall program by the NCBI. The NCBI recommends that people start using the programs of the **blast+** package instead. **On Bio-Linux, this means using the rpsblast+ program.**  **Please note** that on systems that are not Bio-Linux, you will need to find out if the program called rpsblast is from the old blast software or the new blast+ software release. rpsblast+ is a name used in the bio-linux-blast+ package to avoid a naming conflict between the old and new programs.  The programs distributed with blastall include:  - bl2seq - blast2 - blastall - blastcl3 - blastclust - blastpgp - copymat - fastacmd - formatdb - formatrpsdb - impala - makemat - megablast - rpsblast - seedtop - taxblast  The man pages for these provide further details. |
| Homepage | http://www.ncbi.nlm.nih.gov/BLAST/ |
| Remote Documentation | http://www.ncbi.nlm.nih.gov/staff/tao/URLAPI/rpsblast.html      http://www.ncbi.nlm.nih.gov/Education/BLASTinfo/information3.html |
